# A versatile microfluidic device for highly inclined thin illumination microscopy in the moss *Physcomitrella patens*

**DOI:** 10.1038/s41598-019-51624-9

**Published:** 2019-10-23

**Authors:** Elena Kozgunova, Gohta Goshima

**Affiliations:** Division of Biological Science, Graduate School of Science, Nagoya University, Furo-cho, Chikusa-ku, Nagoya, Aichi 464-8602 Japan

**Keywords:** Imaging, Lab-on-a-chip, Plant cytoskeleton

## Abstract

High-resolution microscopy is a valuable tool for studying cellular processes, such as signalling, membrane trafficking, or cytoskeleton remodelling. Several techniques of inclined illumination microscopy allow imaging at a near single molecular level; however, the application of these methods to plant cells is limited, owing to thick cell walls as well as the necessity to excise a part of the tissue for sample preparation. In this study, we utilised a simple, easy-to-use microfluidic device for highly inclined and laminated optical sheet (HILO) microscopy using a model plant *Physcomitrella patens*. We demonstrated that the shallow microfluidic device can be used for long-term culture of living cells and enables high-resolution HILO imaging of microtubules without perturbing their dynamics. In addition, our microdevice allows the supply and robust washout of compounds during HILO microscopy imaging, for example, to perform a microtubule regrowth assay. Furthermore, we tested long-term (48 h) HILO imaging using a microdevice and visualised the developmental changes in the microtubule dynamics during tissue regeneration. These novel applications of the microfluidic device provide a valuable resource for studying molecular dynamics in living plant cells.

## Introduction

Observing protein dynamics in living cells at a single-molecule level provides researchers with valuable data on protein interactions and their function. Although the popularity of live-imaging approaches continues to increase, several techniques remain difficult to use in plants, owing to their slow development and challenging optical properties, such as chloroplast auto-fluorescence and thick cell walls^1,2^. One of the key technologies to monitor cellular events close to the cell surface is total internal reflection fluorescent (TIRF) microscopy. The TIRF method is based on the total reflection of the illumination beam at the boundary of two different reflective indices; for example, between the coverslip and imaging medium^3^. Therefore, TIRF can be applied only when the molecules of interest are very close to the cell surface, which inevitably limits the TIRF use in plant cells surrounded by thick cell walls. Another similar approach is variable angle evanescent microscopy (VAEM), also known as ‘pseudo-TIRF’, oblique illumination fluorescent microscopy or highly inclined and laminated optical sheet (HILO) microscopy, which allows to image events more distant from the cell boundary^4^. TIRF and HILO share certain similarities and the major difference between them is the angle of excitation beam^5^. For both imaging methods, the sample surface has to be flattened against the coverslip. In animal studies, adherent cells provide a natural system for TIRF imaging; alternatively, several coating agents such as fibronectin or poly-l-lysine can be used to attach cells^3^. However, plant samples are usually prepared by covering the excised tissue with an agar block or second coverslip to increase the visible surface^6^. Tissue excision, as well as limited gas exchange, could cause changes in cellular dynamics over time; hence, the plant samples should be observed immediately after preparation. Nevertheless, certain molecules or pathways are active only during particular developmental stages; for example, when the cytoskeleton is dramatically rearranged during cell division^7,8^. Development of long-term HILO imaging of plant cells will help in observing such changes with high resolution.

Microfluidic devices are commonly fabricated from polydimethylsiloxane (PDMS), a biocompatible material suitable for long-term culture conditions^9–12^. PDMS can be moulded into different designs and patterns; for example, a recent study using a microdevice has demonstrated that tip-growing plant cells are able to penetrate extremely narrow 1 µm gaps^13^. In addition, the microfluidic nature of the system enables rapid liquid exchange inside the device during live-cell imaging. Microdevice technology has been routinely applied to supply/washout compounds, such as small-molecule inhibitors for *in vitro* or animal cell research.

In this study, we test and optimise the compatibility of a microfluidic device for HILO imaging in the moss *Physcomitrella patens*, focusing on microtubule dynamics. We demonstrate that our simple, easy-to-use microdevice can be applied in various experiments, such as regular HILO imaging or imaging combined with acute supply/washout of drug compounds. Moreover, we were able to perform a long-term (48 h) HILO imaging using a microdevice to visualise the developmental changes in the cortical microtubules array during tissue regeneration.

## Results

### Severe space restrictions can cause subtle changes in microtubule dynamics in protonema cells

**Figure 1:**
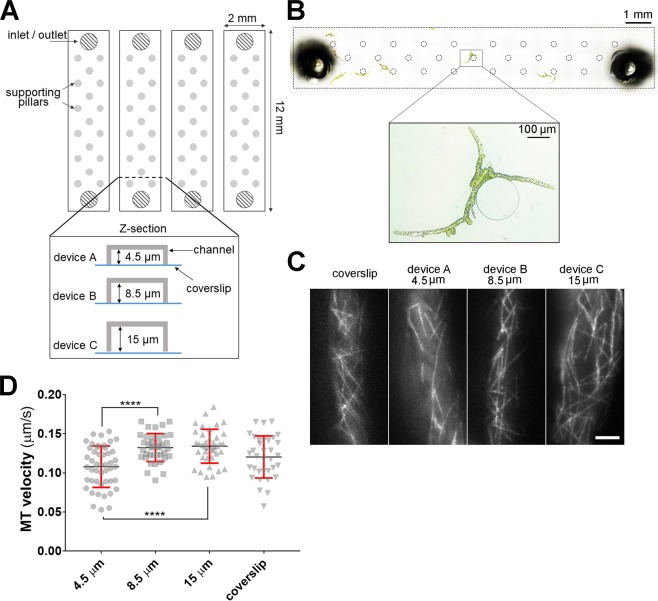
Optimising microfluidic device for HILO live-cell imaging. (**A**) Schematic drawing of the microdevice used for protonema culture and HILO imaging. Three channel depths: 4.5, 8.5, and 15 µm were tested. **(B)** Bright-field image of the single channel (15 µm device) with protonema cells cultured for 3 d. Channel borders and supporting pillars are represented by dotted black lines. **(C)** Representative images of microtubules HILO imaging obtained in four different setups: coverslip sample, 4.5 µm device, 8.5 µm device or 15 µm device. Scale bar = 10 µm. **(D)** Microtubule velocity (growth rate) slightly decreased in the 4.5 µm channels (*n* = 10, 9, 8 and 6 cells for 4.5, 8.5, 15 µm microdevice and coverslip samples, respectively; mean ± SD; ****p < 0.0001 one-way ANOVA).

We used a simple microfluidic device that includes four independent channels (Fig. 1A). This device can be mounted either on a coverslip, for short-term imaging, or on a glass-bottom dish, for long-term imaging. In this study, we used a popular model plant *Physcomitrella patens*^14^, known for its regeneration ability^15,16^. Monthly cultured *P. patens* colonies mostly consist of three tissue types: the protonema comprising tip-growing stem cells, leafy shoots known as gametophores and rhizoids. Diameter of the protonema cells cultured in the agar layer on the glass-bottom dish^17^ was 21.5 ± 2.39 µm (*n* = 16); thus, we hypothesised that the cells grown in shallow channels would be naturally flattened against the coverslip, providing a setting for HILO imaging. We tested three versions of the device with different channel depths: 4.5, 8.5, and 15 µm to determine the optimal conditions (Fig. 1A). On the basis of the average diameter of protonema cells, we considered 4.5, 8.5, and 15 µm depth as severe, average, and mild space restrictions, respectively.

Initially, we injected small clusters of protonema cells, expressing microtubule marker GFP-α-tubulin^18^, into the device and allowed them to regenerate for 3–4 d (Fig. 1B). To test if culturing cells in shallow channels affected cell viability, we performed a propidium iodide (PI) staining. PI is a membrane impermeant dye that stains only cell walls in living plant cells or cytoplasmic contents in non-viable cells^19^ (Supplemental Fig. 1A). We supplied the PI stain at a final concentration 70 µg/mL at 0, 1, 24 and 48 h of cell culture in the microdevice. Notably, we did not observe changes in the viability or growth defects, even in the shallowest 4.5 µm device (Supplemental Fig. 1B).

Next, we investigated how growing cells in shallow channels affected cell shape by using confocal Z-stack imaging and subsequent 3D reconstitution of the cell shape. As a control, we used moss cells cultured for 5 d on the glass-bottom dish as described before^17^. Protonema cells cultured in the microdevice, especially in the 4.5 and 8.5 µm deep channels, were flattened compared to the control (Supplemental Fig. 1C,D, Supplemental Movie 1). Interestingly, the cell diameter based on orthogonal Z views appeared slightly larger than the respective channel height (Supplemental Fig. 1C). We speculate that plant cells, surrounded by the rigid cell walls, could deform PDMS channels, as also observed in the earlier study^13^.

Next, we performed HILO imaging of microtubules in regenerated cells. As a reference, we imaged a freshly prepared sample of protonema cells flattened between two coverslips (Fig. 1C; Supplemental Movie 2). Although a previous report demonstrated that protonema cells are able to grow in extremely narrow channels^13^, it remained unclear if the space restrictions affect the cellular processes. We quantified microtubule growth velocity (Fig. 1D), and found that protonema cells in the 4.5 µm device, but not in the 8.5 or 15 µm devices, show a slight decrease (0.108 ± 0.026; 0.1324 ± 0.018 and 0.134 ± 0.021 µm/s, respectively [*n* = 10, 9, 8]). Interestingly, we also did not notice any advantages in using the 4.5 and 8.5 µm devices in terms of imaging quality or visible cell surface area (Fig. 1C; Supplemental Movie 2). Thus, we chose the device with 15 µm channel depth to conduct further experiments.

### Development of the robust washout protocol using a microdevice

Microfluidic devices have been successfully used for introducing compounds and subsequent washout in bacteria and animal cells^20–22^; however, unlike individual bacteria or animal cells, plant cells are connected by cell walls and form irregular shaped clusters (e.g. Figure 1B), that can partially or completely block the liquid flow in the channels. Although it is possible to digest the cell wall and isolate single cells (protoplasts), the cell wall plays an important role in plant cell physiology^23^. Therefore, we aimed to develop a washout protocol without isolating the protoplast.

The device setup for introducing compounds/washout experiments is presented in Supplemental Fig. 2A (sectional diagram) and 2B (a macroscopic photo). In brief, one inlet hole of the microfluidic channel was connected to a syringe pump and the second inlet hole was covered with a drop of growth medium with or without the compound. Initially, we tested the washout efficiency in the channels with growing protonema cells by measuring the fluorescent intensity during introducing/washing the fluorescent dye AlexaFluor 488 at a final concentration of 0.5 µM (Supplemental Fig. 2C,D; Supplemental Movie 3). We also measured the background fluorescent levels (Supplemental Fig. 2D ‘background’) prior to introducing AlexaFluor 488 as the reference for washing efficiency. In each experiment, we observed that the fluorescent intensity reached the background level within 90 s (*n* = 8) after start of perfusion (Supplemental Fig. 2D). These results indicate that our washout protocol is robust and efficient even with the protonema cells growing in the device. Noteworthy, excessive growth of protonema cells cultured for 6 d in the microdevice partially blocked the liquid flow in the channel, which in turn affected the washout efficiency (Supplemental Movie 4). Thus, long-term culture of protonema cells should be avoided in order to perform robust washout experiments.

### Microtubule nucleation assay

**Figure 2:**
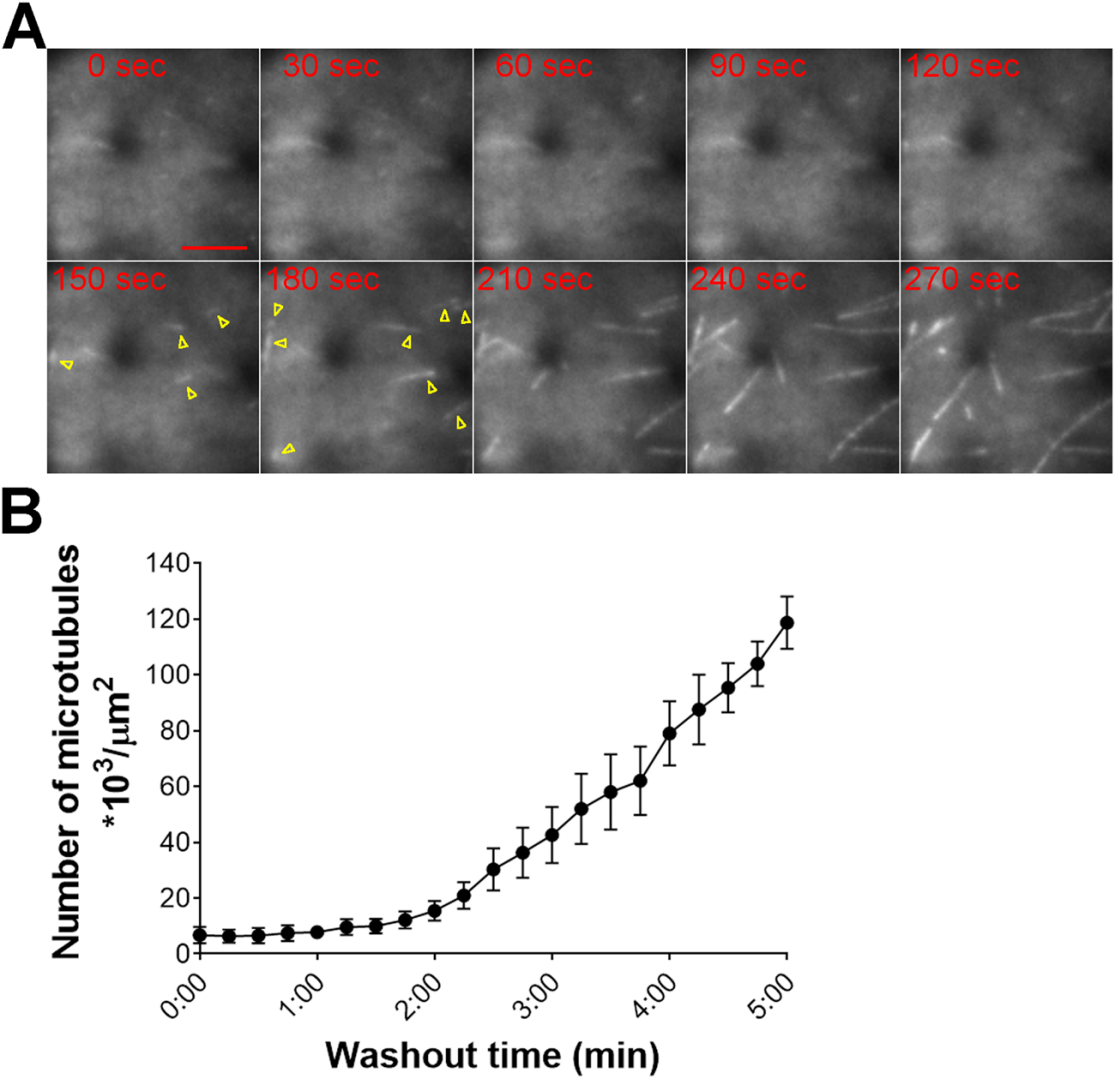
Microtubule nucleation assay in the microdevice. **(A)** Microtubule nucleation observed after oryzalin washout. New microtubules are represented by yellow arrowheads. Oryzalin washout commenced at time 0. Scale = 5 µm. See also Supplemental Movie 5. **(B)** Microtubule density (number of microtubules per area µm^2^) was manually evaluated every 15 s from the washout commencement (*n* = 6, mean ± SEM).

To test if the liquid flow in the channel moves the cells and affects the imaging quality, we conducted a microtubule nucleation assay^24^, using a high-magnification TIRF lens. We used oryzalin treatment at a final concentration of 20 µM for ≥5 min to depolymerise microtubules (Fig. 2A; Supplemental Movie 5). Next, we washed out oryzalin with the fresh culture medium and observed microtubule re-growth for ≥120 s (*n* = 6) after commencing perfusion (Fig. 2A,B; Supplemental Movie 5). The acquired images were of high quality and the observed cells did not drift during imaging. Furthermore, the microtubule density and timing of microtubule nucleation after drug removal (Fig. 2B) were comparable with the previously published results in the protonema cells^24^. Thus, our microfluidic device can be used for washout experiments combined with high-resolution imaging.

### Observing cytoskeleton behaviour in development

**Figure 3:**
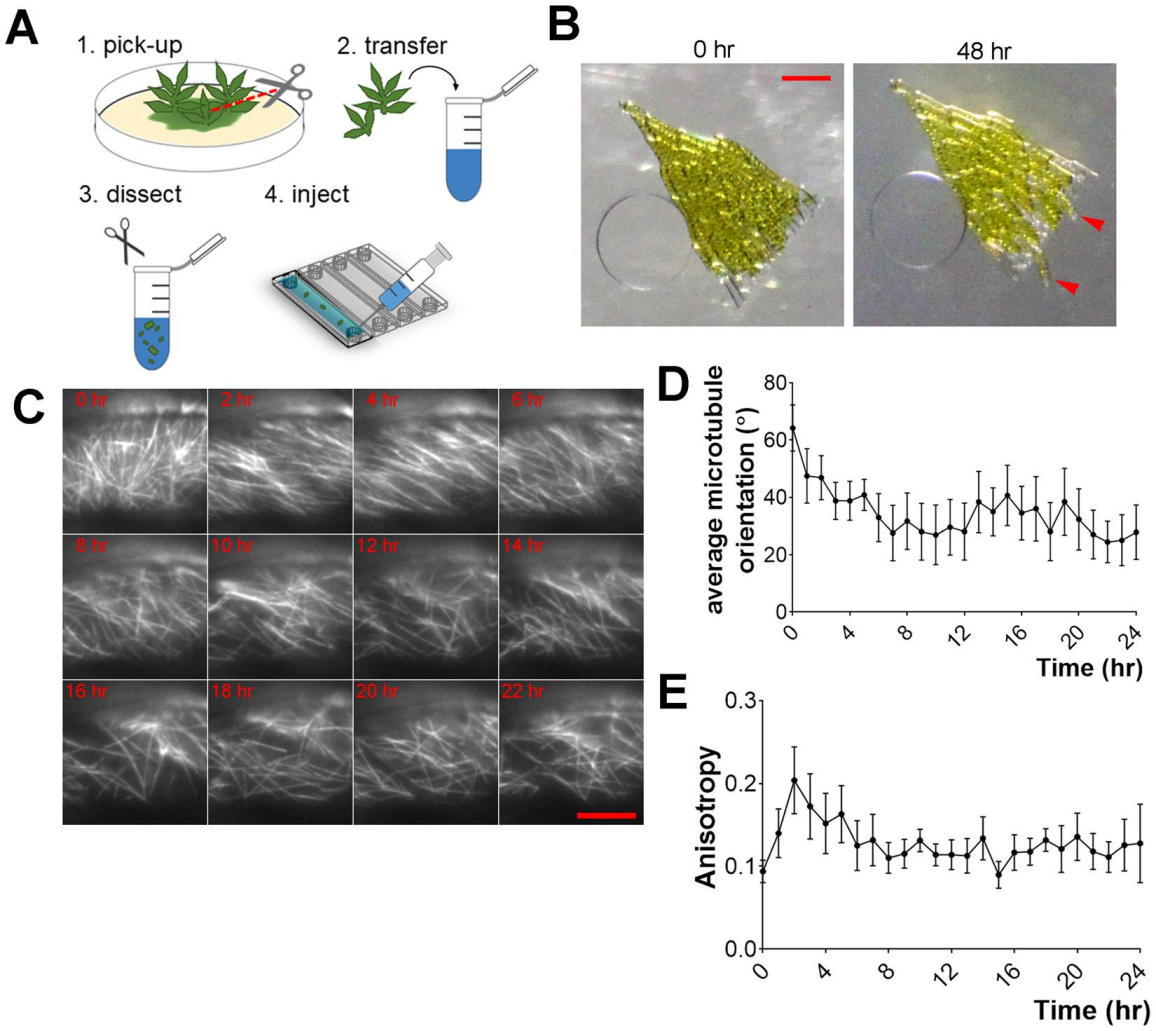
Cytoskeleton remodelling during gametophore regeneration observed with long-term HILO imaging. **(A)** Flowchart for gametophore excision and injection in the microfluidic device. **(B)** Representative image of excised gametophore cells injected in the microdevice and protonema regeneration (red arrowheads) after 48 h. Sample was maintained in 1-min dark/4-min light conditions for imaging. Scale = 100 µm. See also Supplemental Movie 6. **(C)** Representative images of microtubule dynamics in excised gametophore cells. Gradual disassembly of cortical microtubule arrays can be seen (e.g. 4 and 12 h). Scale = 10 µm. See also Supplemental Movie 7. **(D)** Graph of average microtubule orientation (angle) during 24 h time-lapse (*n* = 8, mean ± SEM). Measured every 1 h with FibrilTool ImageJ plugin. **(E)** Graph of anisotropy (score between 0 and 1) changes during 24 h time-lapse (*n* = 8, mean ± SEM). Measured every 1 h with FibrilTool ImageJ plugin.

Next, we tested if the present version of the device is suitable for long-term HILO imaging of developmental changes in microtubule arrangement. Some gametophore cells undergo reprogramming and become protonema cells^15^ after excision (Supplemental Movie 6). We speculated that this process would be accompanied by changes in cytoskeletal dynamics, as the microtubule organisation drastically differs between these two cell types. In gametophore cells, microtubules form two-dimensional cortical arrays^25^ typical for majority of vascular plant cells^26^; in contrast, in protonema cells, they are distributed in the cytoplasm in a 3D manner, forming the so-called endoplasmic microtubule network^24,27^. We performed long-term (48 h) HILO imaging to monitor changes in microtubule organisation in the excised gametophore cells injected into the microfluidic device (Fig. 3A,B; Supplemental Movie 6). We observed cortical microtubule arrays gradually disorganising, and tip growth and phragmoplast formation in some cells (Fig. 3C; Supplemental Movie 7). Furthermore, we estimated changes in the microtubule network by calculating the average microtubule orientation and anisotropy over time (Fig. 3D,E) using FibrilTool plugin^28^. Average microtubule orientation changed from being perpendicular to longer cell axis (64° ± 22.8 at time 0, *n* = 8) to more parallel (30° ± 26.8 after 12 h). Anisotropy score (Fig. 3E) ranging from 0 (purely isotropic arrays) to 1 (purely anisotropic arrays) reflects the alignment of microtubules. For instance, anisotropy values for parallel microtubule arrays, such as cortical microtubules, typically range between 0.2–0.3^28–30^. In our experiments, the initial values for anisotropy were lower than expected (0.09 ± 0.04 at time 0) and recovered in 3 h (0.2 ± 0.11). Later, anisotropy scores gradually decreased, until they reached similar values for protonema cells (0.12 ± 0.04 after 12 h vs. 0.17 ± 0.06, *n* = 8 for excised gametophore cells and *n* = 7 for protonema cells, respectively). Interestingly, we observed that microtubule organisation changed in all gametophore cells, regardless of whether regeneration was observed in protonema cells.

A potential challenge in high resolution long-term imaging is maintaining the sample position. For instance, in our setting we detected sample drift along the X- (0.0019 ± 0.00192 µm/min) and Y-axes (0.002 ± 0.00249 µm/min) by measuring distance between cell positions (*n* = 6) at the first and last time frames. Since there was no liquid perfusion, we interpret that the detected changes are attributed to the stage drift caused by temperature changes, mechanical vibrations in the microscope, etc^31,32^. The stage drift can be variable depending on the microscope setup; thus, optimising microdevice imaging conditions to reduce this type of drift has to be done empirically in each laboratory.

## Discussion

Over the past few years, microfluidic technology is slowly gaining popularity among plant researchers^2^. Microfluidics has been successfully applied to study root growth^10^ and interaction with bacteria^33^, pollen tube attraction^34^, embryo live-cell imaging^35^, and protoplast fusion^36^. Previously, we have applied microfluidic technology to perform short-term HILO imaging^37^. In the present study, we conducted additional tests to determine the optimal channel height for HILO and developed new applications of the microfluidic device, such as robust washout and long-term imaging protocols. By testing several versions of the microdevice, we found that 15-µm-deep channels are sufficient to increase the visible cell surface for HILO imaging. Furthermore, we analysed the microtubule growth rate (velocity) as a representative parameter of microtubule dynamics^38^ and demonstrated that mild space restrictions did not cause significant changes in the microtubule growth rate. We suggest that the 15-µm-deep microdevice can be applied to live-cell imaging of moss *P. patens* in conditions close to natural. Another study developed a microdevice with a 30-µm-deep chamber for long-term culture of *P. patens* to monitor development, including gametophore formation^9^. However, we speculate that this device is not applicable for TIRF or HILO imaging, because the channel depth exceeds the cell diameter.

One of the major applications of microfluidics in cell biology is precise spatiotemporal control of liquids in the channels. It can be used to supply fresh growth medium, create chemical gradients, or supply/remove compounds. The latter application was tested in the present study. We demonstrated that washout is robust and efficient with a few clusters of protonema cells cultured in the channels for 2–3 d. Importantly, we never observed cells drifting during liquid perfusion; therefore, high-resolution imaging could be performed while introducing/washing compounds (e.g. microtubule nucleation assay, Fig. 2). Since plant cells can be cultured in the microdevice for several days, long-term drug treatment combined with imaging is also feasible; however, washout efficiency might be less effective (Supplemental Movie 4). Although only HILO imaging was tested in this study, we suggest that our microdevice can also be used for washout assays during high-resolution confocal microscopy, for instance, to image cell division or organelle movements.

Furthermore, we confirmed that the microfluidic device enables long-term HILO imaging of plant samples. We imaged microtubule reorganisation associated with regeneration of excised gametophore cells^15^ in the microdevice. It is established that cortical microtubule organisation can be affected by internal mechanical cues, such as turgor pressure^39,40^. We speculate that mechanical stress caused by gametophore excision or cell injection in the channels could transiently affect microtubule organisation, which was later recovered (Fig. 3E). Protonema microtubules are distributed in the cytoplasm as a 3D network, whereas, in gametophores, microtubules form more organised parallel arrays with higher anisotropy values^28–30^. We interpret that a slight decrease in anisotropy values over time (Fig. 3E) reflects cell reprogramming to protonema and reorganisation of the microtubule network. Interestingly, we observed changes in the microtubule organisation, such as disassembly of cortical arrays and microtubules reorientation, in all gametophore cells. Previous studies demonstrated that gametophore cells regenerating as protonema produce certain signals, inhibiting protonema regeneration of the surrounding cells^15^. Our observation suggests that all gametophore cells undergo reprogramming and dedifferentiation; however, tip growth and cell division are inhibited in a subset of the cells.

Traditionally, to perform HILO or TIRF imaging, a part of the plant tissue (e.g. root, leaf or protonema cells) is excised and flattened between two coverslips^6,24^. Some cells may be damaged during sample preparation and it is challenging to standardise sample preparation. For instance, in our experience, the viability of protonema cells placed between coverslips was highly variable, ranging from ≤30 min (*n* = 9 out of 26) to ≥2 h (*n* = 17 out of 26). Therefore, we consider using the microfluidic device for HILO imaging as a considerable improvement over the existing methodology. Initially, the cells can be cultured for several days in the microdevice after injection, permitting the adaptation to the new environment. A microdevice is fabricated from a gas-permeable polymer that has been proven to be biocompatible for both plant^9^ and animal cells^11,12^. According to the data of the present study, shallow microfluidic channels are as efficient as coverslips to flatten cells for HILO imaging. Furthermore, long-term HILO imaging can be performed using the microdevice. With minor modifications, such as changing channel depth, the device developed in this study would also be suitable for HILO imaging in other plant model systems (e.g. *Nicotiana tabacum* BY-2 and *Arabidopsis thaliana* culture cells). We anticipate that application of the microfluidic device to HILO microscopy and inhibitor assays will provide valuable insights into plant cell and developmental biology.

## Methods

### Moss culture and transformation

We generally followed the protocols described by Yamada *et al*.^17^. *Physcomitrella patens* culture was maintained on BCDAT (abbreviation stands for stock solutions B, C, D, and ammonium tartrate) medium at 25 °C under continuous light. Transformation was performed with the polyethylene glycol-mediated method, and successful cassette integration was confirmed by PCR^17^ and microscopy. We used the GH line^41^, expressing GFP-tubulin and HistoneH2B-mRFP for microtubule HILO imaging and washout experiments. To observe cortical microtubules in the gametophore, we created a new line, namely GGH, comprising the GCP4 promoter-GFP-tubulin and HistoneH2B-mRFP. For the cell viability assay, we used cell lines expressing only GCP4promoter-GFP-tubulin. The *P. patens* lines used in this study are described in Supplemental Table 1.

### Plasmid construction

Vectors and primers used in this study are described in Supplemental Table 2. The GCP4 promoter-GFP-tubulin vector, assembled with In-Fusion enzyme (TAKARA Clontech), contains eGFP-PpTuA^18^ under endogenous GCP4 promoter (2 kb upstream of the GCP4 gene; accession number Pp3c14_20420, Phytozome) and G418 resistance cassette for selection and homologous recombination sites targeting homeobox 7 (hb7) gene locus^42^. For HistoneH2B-mRFP, we used a previously created vector pGG616^41^, comprising human histone H2B-mRFP fusion gene, directed by the E7113 promoter^43^.

### Microdevice fabrication

The mould for the poly-dimethylsiloxane (PDMS) device was prepared by spin-coating negative photoresist (SU-8 3005 for a 4.5- and 8.5-µm-deep device, 3010 for 15 µm deep device; Microchem Corp.) on a silicon wafer. Maskless lithography system (DL-1000; Nano System Solutions, Inc.) was used to create microchannel designs in the photoresist layer. To construct the PDMS devices, a pre-polymer mixture of PDMS (Sylgard 184; Dow Corning) was prepared by mixing the elastomer base and curing agent at a ratio of 10:1 and pouring it onto the mould. After degassing for 30 min, the mould was placed in an oven (65 °C) for 80 min. The inlet to the microchannel was created using a biopsy punch (1.5 mm diameter; Harris Uni-Core). The PDMS device and a coverslip (Matsunami, glass thickness 0.16–0.19 mm) or glass bottom dish (Matsunami; dish diameter 35 mm; glass diameter 27 mm; glass thickness 0.16–0.19 mm) were both exposed to air plasma for 50 s, pressed together, and heated for 2 h at 70 °C for irreversible bonding of the PDMS layer on the glass surface.

### Introducing moss in the microdevice and viability assay

Microdevice inlet holes were connected to the small pieces of polyethylene tubing (I.D. 0.76 mm; O.D. 1.22 mm; Intramedic). The microdevice was exposed to a vacuum for 15–20 min and immediately filled with BCDAT liquid medium^17^ using a 1 mL syringe with a needle (Terumo, 0.80 × 38 mm). This step helps to prevent air bubbles in the channels. Next, the microdevice was sterilised under UV (2000 × 100 µJ/cm^2^; UVP Crosslinker CL-1000, AnalytikJena).

Protonema cells of *P. patens* were grown on cellophane for 5–7 d^17^. We collected the cells from approximately 1/16 of a standard Petri dish and sonicated in 5 mL of BCDAT liquid medium^17^ (without agar). The sonicated cells were filtered through 50 μm nylon mesh to isolate small cell clusters (typically 1–4 cells), and the flow-through fraction was gently injected in the microdevice. A microdevice with protonema cells was submerged in BCDAT medium to prevent liquid evaporation from the channels; the dish was sealed with parafilm and left for 3–4 d at 25 °C under continuous light.

For the viability assay, we prepared the sample as described above, except for filtering through 50 μm nylon mesh. Cells were cultured for 0, 1, 24, or 48 h as described above. PI solution in BCDAT medium at final a concentration of 70 µg/mL was gently injected in the channel using a 1 mL syringe, just prior to image acquisition.

For gametophore imaging, we picked several gametophores from moss colonies cultured on the BCDAT plates, dissected them into small pieces using scissors in the BCDAT liquid medium and injected in the microdevice using a 1 mL syringe (see Fig. 3A). Imaging was conducted immediately after sample preparation. We recommend using young gametophores, containing 3–4 leaves, for this experiment.

### Introducing compounds/washout experiments

The sonicated cells were filtered through 50 μm nylon mesh to isolate small cell clusters (typically 1–4 cells), and the flow-through fraction was gently injected in the microdevice. The microdevice with protonema cells was submerged in the BCDAT medium to prevent liquid evaporation from the channels; the dish was sealed with parafilm and left for 2–3 d at 25 °C under continuous light. We do not recommend culturing cells for more than 3 d as excessive cell proliferation can block the channels and affect washout efficiency.

One access hole (outlet) was connected to the syringe pump (YMC, YSP-202) using polyethylene tubing (I.D. 0.76 mm; O.D. 1.22 mm; Intramedic) and the second access hole (inlet) was covered with a small drop of BCDAT medium with or without drug (see Supplemental Fig. 2A,B). As the BCDAT medium may contain inorganic salt precipitates, we passed it through 20 µm filter just prior to the experiments. The remaining holes were covered with small pieces of scotch tape to prevent liquid cross-contamination. The device was then mounted on the microscope stage and additionally fixed with a scotch tape. Introducing compounds/washouts were performed in the ‘withdraw’ pump mode at a stable flow rate of 10 µL/min. Every 2–3 min, liquid was supplied on top of the inlet hole using a 20 µL pipette. Notably, the connecting tubing may damage the cells located near the outlet hole; therefore, we suggest to image cells growing in the middle of the channel.

### Live-imaging microscopy and data analysis

Preparation of the coverslip samples was performed as previously described^24^. Epifluorescence microscopy to test the washout efficiency and cell viability was conducted with Nikon’s Ti microscope (10 × 0.45-NA lens), equipped with an electron-multiplying charge-coupled device camera (Evolve; Roper). The HILO imaging was performed with a Nikon Ti microscope with a TIRF unit, a 100 × 1.49 NA lens and EMCCD camera (Evolve; Roper). All imaging was performed at 24–25 °C in the dark, except for the gametophore regeneration assay that requires light (4-min light/1-min dark cycle). The microscope was controlled by the Micro-Manager software and image data were analysed with ImageJ. We used FibrilTool ImageJ plugin^28^ to calculate the average orientation of microtubules and anisotropy in the given region of interest (ROI) from raw images. Microtubule density in the microtubule nucleation assay was evaluated manually.

For 3D reconstitution, images were acquired using the Nikon Ti microscope (40 × 1.30-NA lens) equipped with the spinning-disk confocal unit CSU-X1 (Yokogawa) and an electron-multiplying charge-coupled device camera (ImagEM; Hamamatsu) and 3D reconstitution from Z-stack was performed with Vaa3D software^44^. The microscope was controlled by the NIS elements and image data were analysed with ImageJ. For graphs and statistical analysis, we used GraphPad Prism.

## Supplementary information

Supplementary Information

Supplemental Movie 1

Supplemental Movie 2

Supplemental Movie 3

Supplemental Movie 4

Supplemental Movie 5

Supplemental Movie 6

Supplemental Movie 7

## Data Availability

The authors confirm that the data supporting the findings of this study are available within the article and/or its supplementary materials.
