# Supplementary material for: A versatile microfluidic device for highly inclined thin illumination microscopy in the moss Physcomitrella patens
Source: Sci Rep. 2019 Oct 23;9:15182. doi: 10.1038/s41598-019-51624-9 (PMC6811556; doi:10.1038/s41598-019-51624-9)
Supplement: Supplementary file 1 — Supplementary Information [file 41598_2019_51624_MOESM1_ESM.docx]

**Supplementary Information**

for the article

**A versatile microfluidic device for highly inclined thin illumination microscopy in the moss *Physcomitrella patens***

Kozgunova Elena*, Gohta Goshima

Division of Biological Science, Graduate School of Science, Nagoya University, Furo-cho, Chikusa-ku, Nagoya, Aichi 464-8602, Japan

^*^Correspondence should be addressed to kozgunova@gmail.com

Phone & Fax: +81 52-788-6174


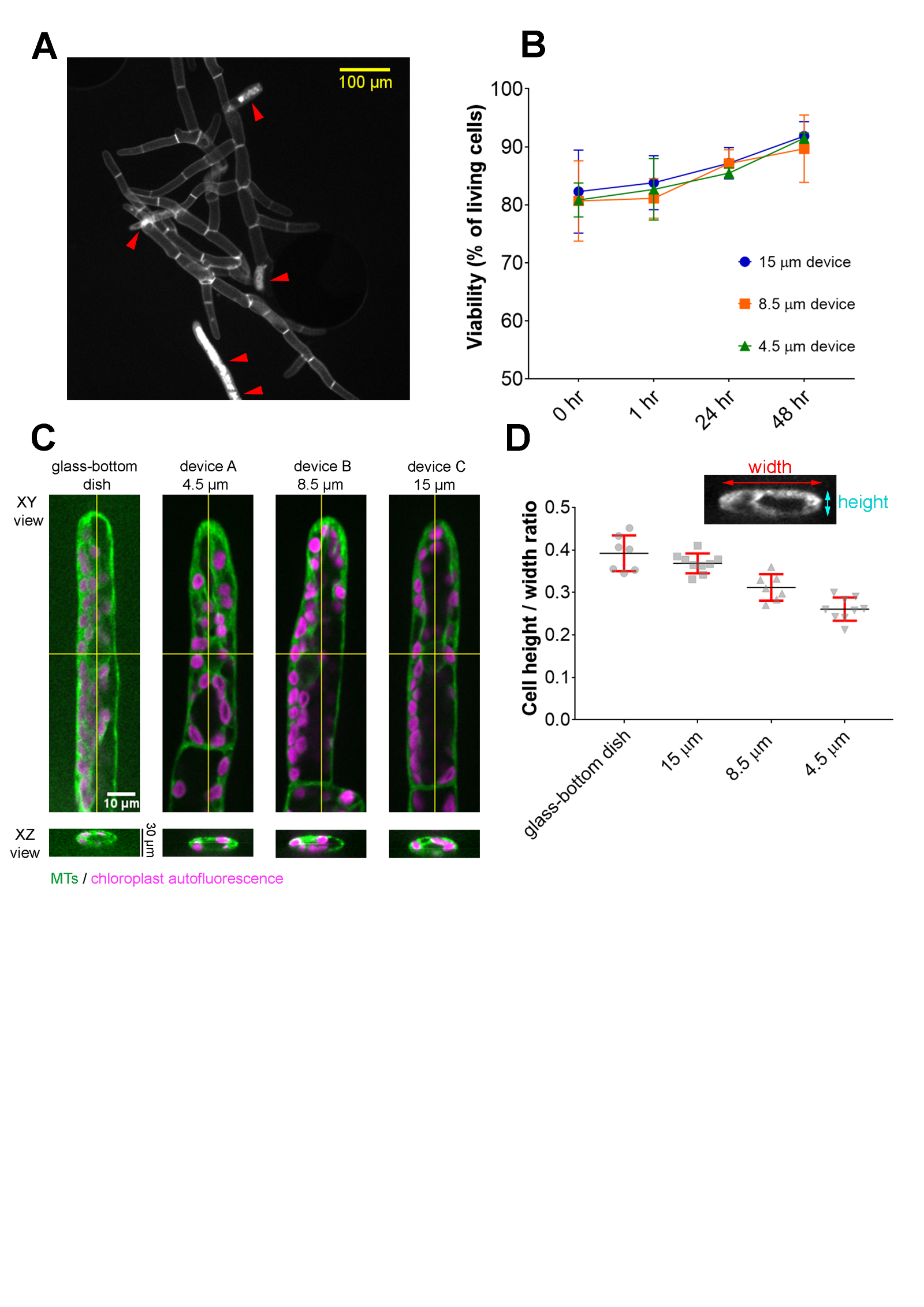


**Supplemental Figure 1. Protonema cell size and viability in the microdevice.**

**(A)** Representative image of propidium iodide (PI) staining at a final concentration of 70 µg/mL in the 15 µm microdevice. Red arrowheads represent dead cells with bright cytoplasmic signals. Bar 100 µm. **(B)** Cell viability (percentage of living cells) of protonema cells cultured in the three height versions of the microdevice for 0, 1, 24 and 48 h. PI solution at a final concentration of 70 µg/mL was introduced just prior to the cell count. More than hundred cells have been counted in each of the three independent experiments (mean ± SD plotted). **(C)** Representative images of XY and XZ (orthogonal Z view) views of protonema cell cultures on the glass-bottom dish or in the microdevices with different channel depths. **(D)** Protonema cells height/width ratio measured from orthogonal Z-views. Decrease in the ratio reflects smaller cell height in the 4.5 and 8.5 µm channels (mean ± SD).


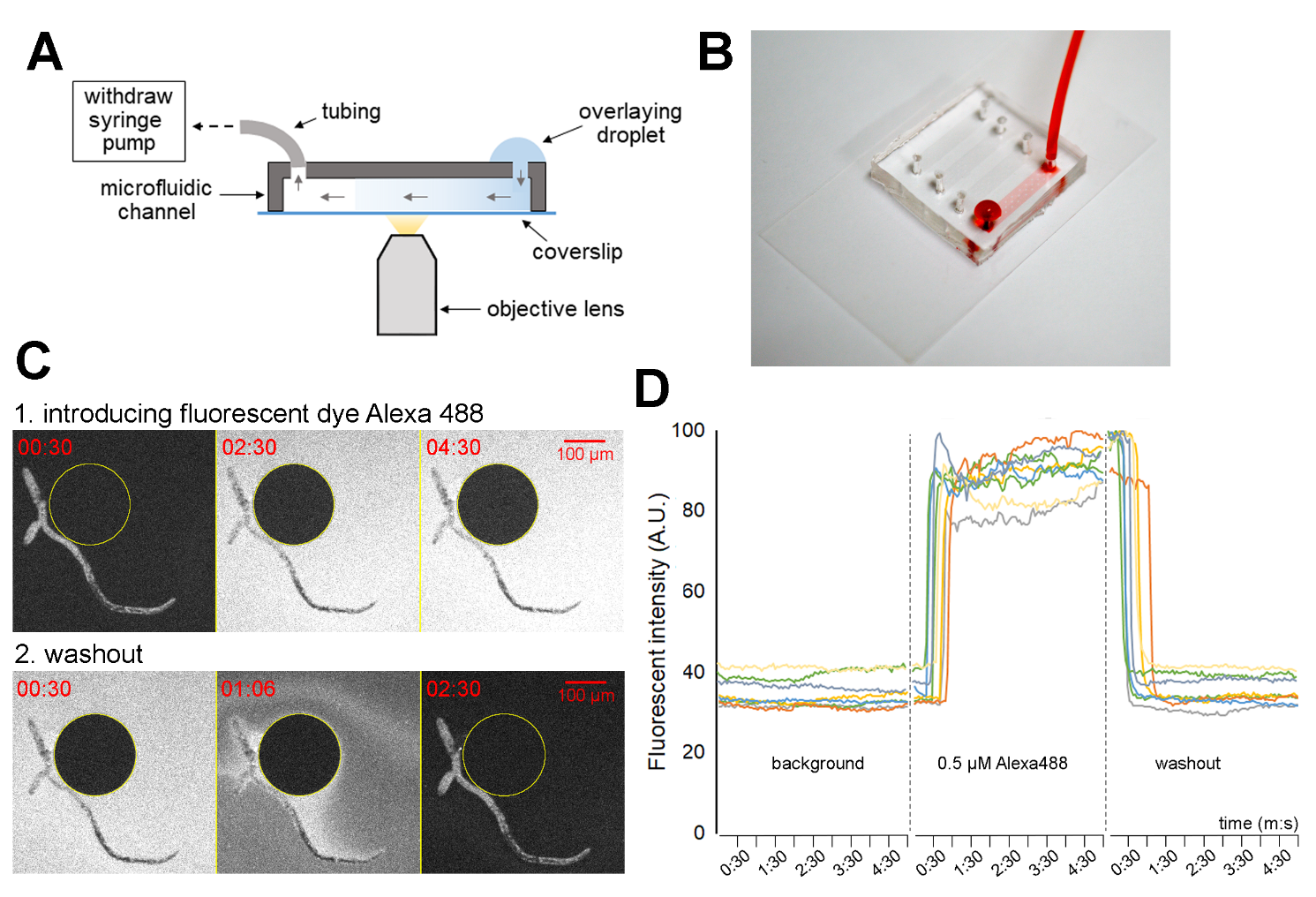


**Supplemental Figure 2. Testing washout setting and efficiency with protonema cells.**

**(A)** Sectional diagram of the washout setup (not to scale). Outlet hole of the microfluidic channel was connected to the syringe pump with tubing. Inlet hole was covered with a drop of liquid with/without the compound. **(B)** A macroscopic photo of the washout setup. Channel is filled with red dye for illustration purposes. **(C)** Representative images of introducing/washing out fluorescent dye AlexaFluor 488 at a final of concentration 0.5 µM. See also Supplemental Movie 3. **(D)** Changes in average fluorescent intensity (A.U.) in the microfluidic channels before introducing the dye (background), during dye perfusion (0.5 µM AlexaFluor 488) and washout. Fluorescent intensity was measured in five randomly selected areas (25 × 25 pixels) close to the protonema cells at each time point; mean intensity is plotted on the graph. Each line represents an individual experiment (*n* = 8).

| **Supplemental Table 1. Transgenic moss lines used in this study** | | | |  |  |
| --- | --- | --- | --- | --- | --- |
| Line# | Clone# | Name | Plasmids | Resistance | Reference |
| 0002 | 3 | **GH** (**G**FP-tubulin/**H**2B-RFP) | GFP-tubulin pGG616 | Zeocin, G418 | Nakaoka et al., 2012 |
| 0686 | 2 | GCP4promoter-GFP-tubulin | pSY100 | G418 |  |
| 0704 | 15, 30 | **GGH** (**G**CP4promoter-**G**FP-tubulin/**H**2B-RFP) | pSY100 pGG616 | Zeocin, G418 |  |

| **Supplemental Table 2. Primers and plasmids used in this study** | | |  |  |  |
| --- | --- | --- | --- | --- | --- |
| Plasmid | Gene/fragment | 5` primer | 3` primer | Selection marker | targeting site |
| pSY100 | GCP4 promoter | ACGAATTCGAGCTCGGGAAGGTTCCAAGAGAAGAAATTCA | GCCCTTGCTCACCATCGCGCAACTCTTTGCTACAC | G418 | hb7 |
|  | eGFP-tubulin | GCAAAGAGTTGCGCGATGGTGAGCAAGGGCGAGGAGCTG | tggccaaatcggccgTCAGTAGTCGTCGTCCTCCGGACC |  |  |
| **Genotyping PCR** | |  |  |  |  |
| pSY100 |  | TGGCACTCCGCTCGGCACAAC | ATGTCGGACCTCTCCGATTGC |  | hb7 |
| pGG616 |  | ACATATGCATGTATCAATCCAATGACTTGG | AACATGGTGCAGCAGATATCTCTGTCC |  | BS213 |

**Supplemental Movie 1.** Reconstituted 3D image of protonema cells with GFP-tubulin (green) and chloroplast autofluorescence (magenta). The 3D image was reconstructed from 31 Z-stack images acquired at 1 µm interval. XY scale bar 20 µm, Z scale bar 30 µm.

**Supplemental Movie 2.** HILO imaging of microtubule network using different set-ups: coverslip sample, 4.5, 8.5 or 15 µm deep microfluidic channels. Imaging was conducted in the protonema cells expressing GFP-tubulin. Images depict a single focal plane acquired every 3 s.

**Supplemental Movie 3.** Introducing fluorescent dye AlexaFluor488 *(left)* and subsequent washout *(right)*. Position of the supporting pillar is represented by the yellow circle. Protonema cells are expressing GFP-tubulin. Images depict a single focal plane acquired every 3 s.

**Supplemental Movie 4.** Excessive growth of plant cells can disturb liquid flow in the channel, as was demonstrated by inefficient washout of fluorescent dye AlexaFluor488. Channel wall and supporting pillar are represented by yellow lines. Protonema cells are expressing GFP-tubulin. Images depict a single focal plane acquired every 3 s.

**Supplemental Movie 5**. Microtubule nucleation assay using microdevice, *(left)* introducing microtubule-depolymerising drug oryzalin at a final concentration of 20 µM, *(right)* oryzalin washout and microtubule regrowth. Imaging was conducted in protonema cells expressing GFP-tubulin. Images depict a single focal plane acquired every 3 s.

**Supplemental Movie 6.** Regeneration of excised gametophore cells to protonema in the microdevice. Imaging was conducted in protonema cells expressing GFP-tubulin under native GCP4 promoter (microtubules, green) and HistoneH2B-mRFP (nucleus, magenta). Images depict a single focal plane acquired every 5 min.

**Supplemental Movie 7.** HILO imaging of microtubule network in excised gametophore cells during regeneration. Imaging was conducted in protonema cells expressing GFP-tubulin under native GCP4 promoter. Phragmoplast can be seen at the 25 h 40 min time-frame. Images depict a single focal plane acquired every 5 min.
